# Protective effects of vitamin D_3_ on fimbrial cells exposed to catalytic iron damage

**DOI:** 10.1186/s13048-016-0243-x

**Published:** 2016-06-17

**Authors:** Francesca Uberti, Vera Morsanuto, Debora Lattuada, Barbara Colciaghi, Andrea Cochis, Alessandro Bulfoni, Paola Colombo, Giorgio Bolis, Claudio Molinari

**Affiliations:** Department of Obstetrics and Gynecology, Fondazione IRCCS Cà Granda, Ospedale Maggiore Policlinico, Milan, 20122 Italy; Physiology Laboratory, Department of Translational Medicine, UPO - University of Eastern Piedmont, Via Solaroli 17, Novara, 28100 Italy; Department of Biomedical, Surgical and Dental Sciences, Milan State University, via Beldiletto 1, Milan, 20142 Italy

**Keywords:** Fimbrial secretory epithelial cells, Catalytic iron, Epithelial ovarian cancer, Vitamin D_3_

## Abstract

**Background:**

Recently, vitamin D_3_ (1alpha, 25-dihydroxyvitamin D) has shown its capability to take part in many extraskeletal functions and its serum levels have been related to patient survival rate and malignancy of many types of neoplasms, including ovarian cancers. Catalytic iron is a free circulating form of iron that is able to generate reactive oxygen species and consequently to promote a number of cellular and tissutal dysfunctions including tumorigenesis. In fertile women an important source of catalytic iron is derived from retrograde menstruation. Epithelial secretory cells from fimbriae of fallopian tubes are greatly exposed to catalytic iron derived from menstrual reflux and so represent the site of origin for most serous ovarian cancers.

The aim of this study was to assess whether vitamin D_3_ can play a role in counteracting catalytic iron-induced oxidative stress in cells from fimbriae of fallopian tubes.

**Methods:**

The cells, isolated from women undergoing isteroannessiectomy, were treated with catalytic iron 50-75-100 mM and vitamin D_3_ at a concentration ranging from 0.01 to 10 nM to study cell viability, radical oxygen species production, p53, pan-Ras, Ki67 and c-Myc protein expressions through Western Blot, and immunocytochemistry or immunofluorescence analysis.

**Results:**

The pre-treatment with vitamin D_3_ 1 nM showed its beneficial effects that consists in a significant decrease in ROS production. In addition a novel finding is represented by the demonstration that pre-treatment with vitamin D_3_ is also able to significantly counteract tumoral biomarkers activation, such as p53, pan-Ras, Ki67 and c-Myc, and consequently the catalytic iron-induced cellular injury.

**Conclusions:**

This study demonstrates for the first time that vitamin D_3_ plays an important role in preventing catalytic iron-dependent oxidative stress in cultured fimbrial cells. These results support the hypothesis that vitamin D_3_ could counteract carcinogenic changes induced by catalytic iron.

## Background

Recently, the role of 1alpha,25-dihydroxyvitamin D (VitD) has greatly expanded from its classical function of modulator of calcium metabolism and skeletal trophism into a number of extraskeletal functions such as nitric oxide production, antioxidant activity and endothelial proliferation and migration [1–3]. Along with its well-known metabolic functions, VitD has recently shown immunomodulatory and anticancer properties as well [4–7]. As a matter of fact, the importance of serum VitD level as a biomarker for cancer risk was first determined by Garland et al. in 1989 [8]. Moreover, Gorham et al. [9] confirmed the relationship between VitD and cancer, showing that an increase in the serum level of VitD to 34 ng/ml was associated with a 50 % reduction in incidence rates of colorectal cancer.

As regards ovarian cancer, it has been observed that serum concentration of VitD was lower in cancer patients than in the reference group (12.5 ± 7.75 ng/mL *vs* 22.4 ± 6.5 ng/mL) [10]. Moreover, low VitD serum concentration is associated with lower overall survival rate. This fact points out the importance of severe VitD deficiency as a possible cause of highly aggressive ovarian cancer [10]. In addition, VitD treatment suppressed human epithelial ovarian cancer cells migration and invasion in monolayer scratch and transwell assays, as well as the ability to colonize the omentum in an *ex vivo* experimental model. These findings support a role for epithelial VitD receptor (VDR) in interfering with epithelial ovarian cancer invasion [11]. A recent systematic review states that there is no consistent or strong evidence to support the claim made in numerous review articles that VitD exposure reduces the risk for ovarian cancer occurrence or mortality [12]. However, this declaration is in contrast with several human and cell-based studies which show that VitD can induce growth arrest and apoptosis either of tumor cells or of their non-neoplastic progenitors [13, 14]. In addition, it has been demonstrated that other gene targets related to DNA repair and immunomodulation, as well as other cell targets such as the stromal cells and cells of the immune system, may be regulated by VitD, thus contributing to cancer prevention [15]. The molecular mechanisms leading to cancer prevention exerted by VitD have been extensively studied in the last few years in order to identify a possible new therapeutic strategy. A large number of studies have shown that VitD has important anti-proliferative, anti-angiogenic and pro-differentiative effects in a wide range of cancers. These effects are mediated through perturbation of several important signalling pathways mediated through genomic and non-genomic mechanisms [16]. However these effects have no uniform patterns of modulation by VitD across different types of cancer cell lines. It has been hypothesized that the heterogeneous action of VitD may depend on the differentiation status of the cancer cells and VDR expression level, as well as genomic or post-translational modifications of co-activator proteins that are essential for the assembly of the transcriptionally active VDR complex [17].

Catalytic iron (Fe^3+^) is a free circulating iron that is not bound to transferrin or ferritin and is known to generate reactive oxygen species that may have noxious effects on cells and tissues. For example, a number of studies show that high levels of Fe^3+^ may promote atherosclerosis [18], endothelial dysfunction, arterial smooth muscle proliferation and ischemia/reperfusion injury [19, 20]. Blood-deriving Fe^3+^ can accumulate into tissues and cells where its ability to switch from its ferrous oxidation state into its ferric one reversibly makes it very dangerous since free iron can catalyze the formation of free radicals, which can damage molecular components of the cell [21]. In a chronic condition, high concentrations of heme and free iron (Fe^3+^) derived from lysis of red blood cells by macrophages are able to exceed the capacity of ferritin to sequester iron leading to oxidative injury. This mechanism generates oxygen-free radicals leading to numerous carcinogenic DNA mutations or loss, genetic instability, overexpression of specific oncogenes, and downregulation of tumor suppressor genes [22–24]. It has recently been demonstrated that Fe^3+^, derived from menstrual reflux, recently defined as “incessant menstruation” [25], is able to induce an increase in fimbrial cell viability and proliferative capacity and to activate principal oncogenes (p53, pan-Ras, Ki67 and c-Myc). So, it has been confirmed that Fe^3+^ is capable to induce carcinogenic changes and represents the main non-genetic risk factor for ovarian cancer [26]. For this reason, Fe^3+^ can be considered a putative candidate as a transforming agent from normal human fimbrial cells into cancer cells maintaining physiological conditions of the menstrual cycle through oxidative stress and consequent oncogenes activations.

This research was planned to study the role of VitD to prevent oxidative injury induced by Fe^3+^ exposition in primary fimbrial cells culture, because recent studies have hypothesized that fimbrial fallopian tubes are the site where most serous ovarian cancers develop [27, 28], and the cells are subjected, to a constant carcinogenic stimulus represented by Fe^3+^ [26], especially in presence of low levels of serum VitD.

## Methods

### Samples

Thirty-six fresh fallopian tube-derived fimbriae were obtained under written consent from women during isteroannessiectomy for ovarian cancer and benign pathology without comorbidity at the II Department of Obstetrics and Gynaecology of the Fondazione IRCCS Ca’ Granda, Ospedale Maggiore Policlinico (Milan, Italy). All subjects were in premenopause and had not received any type of hormonal or drug therapy for at least 3 months. Approval for this study was granted by the local Human Institutional Investigation Committee.

### Tissue collection

Thirty-six fresh fallopian tube-derived fimbriae tissues were collected during isteroannessiectomy from 18 women and transported to laboratory in sterile falcon containing saline solution (0.9 % w/v solution of sodium chloride in distilled water, S.A.L.F, Cenate Sotto, Bergamo, Italy) supplemented with 10 % penicillin/streptomycin (Sigma, Milan, Italy). The tissue were obtained under written consent at the II Department of Obstetrics and Gynaecology of the Fondazione IRCCS Ca’ Granda, Ospedale Maggiore Policlinico (Milan, Italy). Approval for this study was granted by the local Human Institutional Investigation Committee. All 18 women were in premenopause and had not received any type of hormonal or drug therapy for at least 3 months before isteroannessiectomy for ovarian cancer and benign pathology without comorbidity.

### Primary cell preparation and culture

The isolation of epithelial secretory cells from fimbriae of fallopian tubes (FSEC) has been described in detail in a previous study [26]. Briefly, samples of fimbrial tissues were washed, minced and incubated with Dulbecco’s Modified Eagle Medium (DMEM, Sigma, Milan, Italy) supplemented with 0.1 % type A collagenase, 1 % penicillin/streptomycin and 2 mM L-Glutamine (Sigma, Milan, Italy) for 2-3 h in incubator at 37 °C in agitation and centrifuged at 600xg for 10 min at room temperature (RT). The cell population of each patient have been kept separate and used for the experiments. One experiment was performed on one population obtained from one patient. Before the experiments, the purity of cell culture was verified using a specific marker PAX8. The cells used had passage 1-5 to have a complete cell phenotype.

To study cell viability (MTT test) and ROS production 1x10^4^ cells were plated on 24 well-plates; to perform immunohistochemistry and immunofluorescence studies 0.2x10^4^ cells were placed in CultureSlide (BD, Bedford, MA, U.S.A.) with 4 chambers; to analyze the intracellular pathways through Western Blot analysis the cells were plated on 60 mm culture dish until confluence. Each experiment was performed on 4 to 6 cell population of FSEC to obtained 4 or 6 technical replicates using 50, 75, 100 mM of Fe^3+^.

### Experimental protocol

Each experiment was performed on 4 to 6 FSEC using 50, 75, 100 mM of Fe^3+^ [26] and 1nM VitD (based on dose-response study) [2]. 1nM VitD was able to induce a maximum effect on cell viability of FSEC. This concentration was also verify in other work reported in literature [2]. This VitD concentration can ben considered physiologically and clinically attainable because in humans is comprised between 0.1 nM and 10 nM [29]. Experiments were performed using high doses of Fe^3+^ comparable to those observed in the content of endometriotic cysts [30]. In addition these concentrations were also observed in a previous work on fimbrial cells to be able to mimic carcinogenic changes [26]. FSEC were incubated for two hours in DMEM without red-phenol supplemented with 1 % penicillin/streptomycin, 2 mM L-Glutamine and 0.5 % FBS before and during the treatment. The stimulation with 1nM VitD was maintained alone for 6 days and replicated as pretreatment for 6 days before the stimulation with Fe^3+^ for other 6 days. The time of stimulation of Fe^3+^ was the same used in a previous work [26].

### MTT test

MTT dye (Sigma-Aldrich) was used to determine cell viability. After stimulation the cells were incubated with 1 % MTT dye for 2 h at 37 °C in incubator, as previously described [2, 26].

Then, the medium was removed and the crystals were dissolved in DMSO. Cell viability was measured through a spectrometer (VICTORX3 Multilabel Plate Reader) at 570 nm with correction at 690 nm, and calculated by comparing results to control cells (100 % viable).

### ROS production

The rate of superoxide anion release was used to examine the effects of VitD against the oxidative stress induced by Fe^3+^. The superoxide anion production was measured as superoxide dismutase-inhibitable reduction of cytochrome C, as previously described [2]. Briefly, in all samples (stimulated and untreated), 100 μL of cytochrome C was added and in another one, 100 μL of superoxide dismutase was also added for 30 min in an incubator (all substances from Sigma-Aldrich). The absorbance changes in the supernatants of the sample was measured at 550 nm in a Wallac Victor model 1421 spectrometer (PerkinElmer). The O_2_ was expressed as nanomoles per reduced cytochrome C per microgram of protein, using an extinction coefficient of 21000 mL/cm, after the interference absorbance subtraction [31].

### Western Blot for VDR, PAX8, p53, c-Myc, Ki67 and pan-Ras

FSEC at confluence were washed three times with cold PBS 1x supplemented with 2 mM sodium orthovanadate, and then lysed in ice with Complete Tablet buffer (Roche) supplemented with 2 mM sodium orthovanadate and 50 μM MG132 (Sigma-Aldrich). Thirty-five μg of proteins from each lysate were loaded on 15 or 5 % SDS-PAGE gels and transferred to polyvinylidene fluoride membranes (PVDF, GE Healthcare Europe GmbH, Milan, Italy). They were incubated overnight at 4 °C with specific primary antibody: anti-PAX8 (1:500, Abnova, DBA ITALIA S.r.l., Milan, Italy), anti-VDR (1:200, Santa-Cruz), anti-p53 (1:500, Santa-Cruz), anti-cMyc (1:200, Millipore S.p.A., Milan, Italy), anti-Ki67 (1:500, Santa-Cruz), and anti-pan-Ras (1:500, Santa-Cruz). Protein expression was normalized and verified through ß-actin detection (1:5000; Sigma, Milan, Italy).

### VDR, PAX8, c-Myc, Ki67 and pan-Ras immunocytochemistry in cellular preparation

FSEC cultured in chamber slide as described above were washed three times with cold PBS 1x supplemented with 2 mM sodium orthovanadate, and fixed using a cold fixative solution (3.7 % formaldehyde, 3 % sucrose in PBS 1X) for 20 min at RT. Then the cells were washed twice with cold PBS 1X, permeabilized with cold PBS 1X with cold 0.5 % Triton X-100 on ice at 4 °C for 20 min and then washed with PBS 1X. Then the chamber slides were incubated with 3 % hydrogen peroxide in PBS 1X for 8 min to block endogenous peroxidase activity and then maintained in a blocking solution composed of PBS 1X with 3 % albumin from bovine serum (BSA, Sigma, Milan, Italy) for 1 h at RT. The slides were subsequently incubated overnight at 4 °C with specific primary antibody: 1:150 PAX8, 1:50 VDR, 1:50 c-Myc, 1:50 Ki67 and 1:50 pan-Ras. All these antibodies were diluted in PBS 1X in a humidified chamber, and then incubated for 20 min with diluted biotinylated secondary antibody solution (Dako Italia, Milan, Italy) and then for 20 min with VECTASTAIN® ABC Reagent (Dako Italia, Milan, Italy). Finally the sections were washed, incubated with peroxidase substrate solution until desired stain intensity developed (Peroxidase/DAB, Dako Italia, Milan, Italy), rinsed in tap water, counterstained with Mayer’s hematoxylin and mounted with Bio Mount (Bio-Optika, Milan, Italy). The number of positive cells was calculated as described elsewhere [32]: briefly, 12 different areas (1 mm^2^) randomly selected from each section were taken, and the number of signals was determined using ImagePro 3 software (NIH, Bethesda, US). The results were expressed as a means ± SD (%).

### p53 immunofluorescence in cellular preparation

After the stimulations the cells were fixed using cold buffer PAF for 20 min, washed three times with cold PBS 1X and then permeabilized with cold PBS 1X with 0.5 % Triton X-100 for 20 min at 4 °C. After this time slides were incubated in blocking solution (1 % BSA and 5 % FBS in PBS 1X) for 30 min at RT and treated with p53 specific antibody (1:50, Santa-Cruz) in PBS 1X overnight at 4 °C. The slides were then incubated with fitch-secondary antibodies (1:200, Sigma-Aldrich) in PBS 1X for 1 h in the dark, counterstained with DAPI (1 μg/ml; Sigma-Aldrich) diluted in PBS 1X for 5 min in the dark at RT and finally mounted in Vectashield (D.B.A. Italia). The number of positive cells was calculated as described by Lee et al. [32]: briefly, 12 different areas (1 mm^2^) randomly selected from each section were taken, and the number of signals was determined using ImagePro 3 software (NIH, Bethesda, US). The results were expressed as means ± SD (%).

### Statistical analysis

Results are expressed as means ± SD of at least 4 independent experiments for each experimental protocol. One-way ANOVA followed by Bonferroni post hoc test was used for statistical analysis. The percentage values were compared through Mann-Whitney U test. *P*-value <0.05 was considered statistically significant.

## Results

### Dose-response study, cell viability and reactive oxygen species (ROS) production

**Fig. 1:**
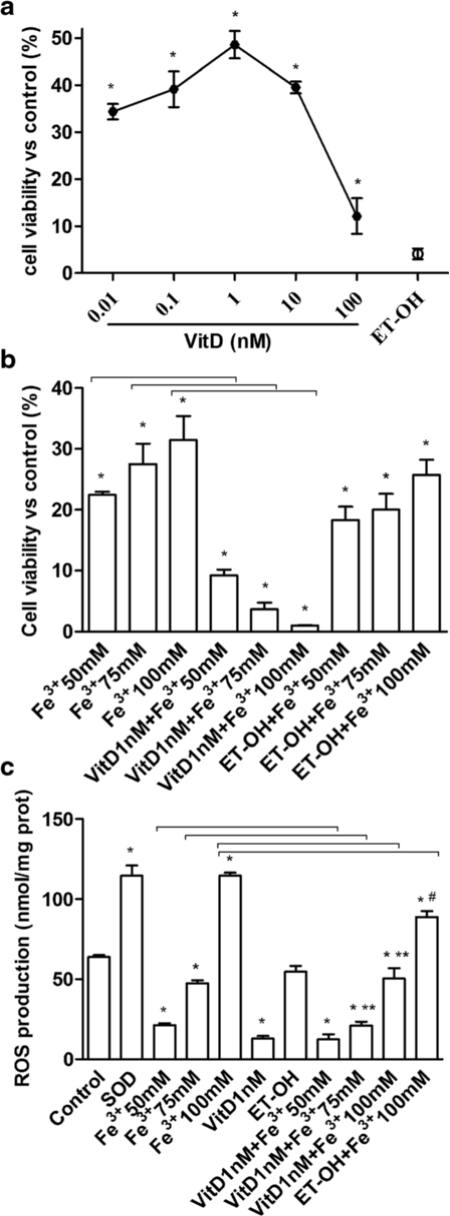
Effects of VitD on cell viability and ROS production. **a**, dose-response study of VitD (0.01-100 nM) on cell viability of FSEC. The results are expressed as means ± SD (%) normalized to control values of 5 technical replicates. * *P* < 0.05 *vs* control. **b**, MTT test performed in FSEC treated with different doses of Fe^3+^ (50-100 mM) alone for 6 days or pretreated with 1nM VitD for 6 days. The results are expressed as means ± SD (%) normalized to control values of 6 technical replicates. ET-OH = ethanol * *P* < 0.05 *vs* control; arrows indicate *P* < 0.05 between Fe^3+^ alone and VitD + Fe^3+^ **c**, ROS production (expressed as Cytochrome C reduced per μg of protein) in FSEC treated with different doses of Fe^3+^ (50-100 mM) alone for 6 days or pretreated with 1nM VitD for 6 days. The results are expressed as means ± SD of 5 technical replicates. ET-OH = ethanol. * *P* < 0.05 *vs* control; ** *P* < 0.05 *vs* VitD; ^#^ *P* < 0.05 *vs* ET-OH; arrows indicate *P* < 0.05 between Fe^3+^ alone and VitD + Fe^3+^

The cell viability induced by stimulation of FSEC with VitD (from 0.01 to 10 nM, dissolved in ethanol), was measured in a dose-response study after 6 days by MTT test. As illustrated in Fig. 1a, the effect of VitD on cell viability by a dose-response study was examined and it was concentration-dependent with a maximum effect at 1 nM concentration after 6 days of stimulation (48.60 ± 2.93 %, *P* < 0.05). This concentration was used for all successive experiments. The effect of the solvent of VitD on FSEC was also tested. In addition the influence of VitD on the effects exerted by Fe^3+^ on cell viability of FSEC was studied. The cells were divided into two groups: one treated with different concentrations of Fe^3+^ for 6 days and one pre-treated with 1 nM VitD for 6 days and then stimulated with the same concentrations of Fe^3+^ (50-75-100 mM). Pre-treatment was also performed using ethanol alone (solvent of VitD). As reported in Fig. 1b, the pre-treatment with VitD was able to counteract the increase on cell viability induced by Fe^3+^ in a dose-dependent manner, and the maximum effects were observed in presence of 100 mM Fe^3+^ (1.033 ± 0.04 %, *P* < 0.05) in respect with Fe^3+^ alone.

The same conditions described before were reproduced to analyze the ROS production in FSEC. In cells treated with Fe^3+^ we observed a significant increase in ROS production in a dose-dependent manner compared with control (Fig. 1c, *P* < 0.05), and the maximum effects was obtained by 100 mM Fe^3+^ (114.6 ± 1.84 Cytochrome C reduced per μg of protein). In FSEC treated with VitD alone a significant reduction in ROS production compared to control was observed (13.08 ± 1.53 Cytochrome C reduced per μg of protein). The pre-treatment with VitD was able to counteract the ROS production induced by Fe^3+^ and this effect was more evident in presence of 75 mM (21.09 ± 2.46 Cytochrome C reduced per μg of protein) and 100 mM Fe^3+^ (50.27 ± 6.59 Cytochrome C reduced per μg of protein) compared with Fe^3+^ alone (47.40 ± 1.86 and 114.6 ± 1.84 Cytochrome C reduced per μg of protein, respectively). These data confirmed previous findings on MTT test, and demonstrate the ability of VitD to prevent the effects of oxidative stress only if it used before the oxidative damage.

### PAX8 and VDR receptor analysis

**Fig. 2:**
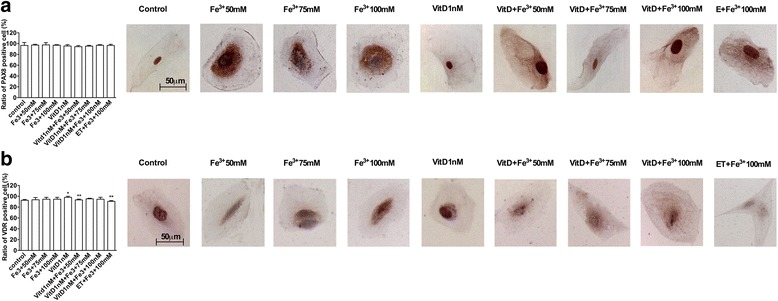
PAX-8 and VDR receptor analysis by immunocytochemistry. On the left the score of positive cells, counted in 12 different areas, and on the right the representative pictures, obtained through microscopy at original magnification of X40, are reported. A, PAX-8 staining: the ratio is reported as mean (SD) (%) of positive cells of 4 technical replicates. The scale reported in the first column on the right is to be considered valid for the same antibody. B, score of positive cells for VDR receptor is reported as mean (SD) (%) of 4 technical replicates. * *P* < 0.05 *vs* control; ** *P* < 0.05 *vs* VitD. The scale reported in the first column is to be considered valid for the same antibody

**Fig. 3:**
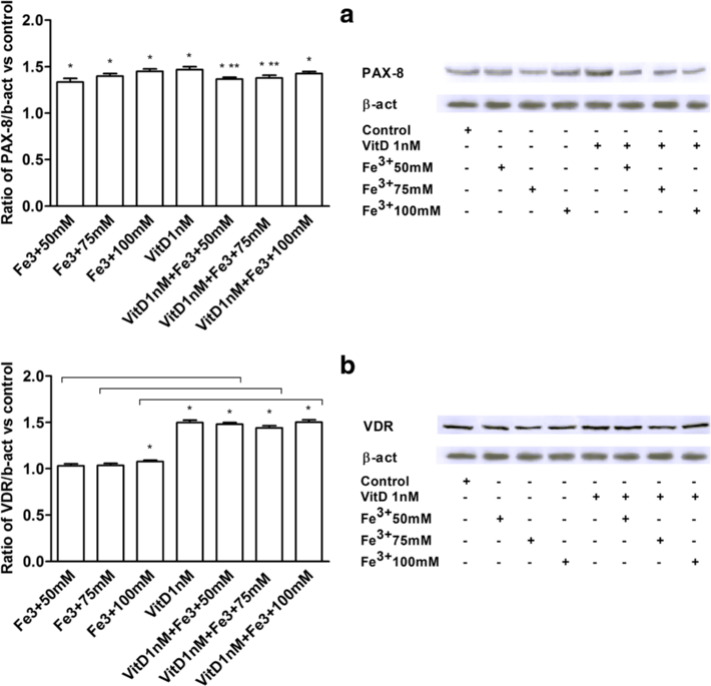
PAX-8 (**a**) and VDR (**b**) studies by Western Blot and densitometric analysis. Protein extracts has been analyzed by immunoblotting with specific antibodies against the indicated proteins. Data are expressed as means of 4 technical replicates performed on FSEC. * *P* < 0.05 *vs* control; ** *P* < 0.05 *vs* VitD; arrows indicate *P* < 0.05 between Fe^3+^ alone and VitD + Fe^3+^

FSEC were tested for specific fimbrial marker PAX8 in immunocytochemistry and Western blot analysis (Figs. 2a and 3a) to verify the efficacy of cell isolation and the preservation during the stimulation with Fe^3+^ and VitD (as showed nuclear/perinuclear staining by immunocytochemistry). The presence of VDR receptor in FSEC were determined to demonstrate the efficacy of VitD in these cells. The presence of VDR receptor was evident in immunocytochemistry (Fig. 2b), in which 98 ± 2 % of FSEC had cytoplasmic-nuclear staining positive and this increase compared to control was also observed by Western blot analysis (Fig. 3b). Indeed the expression was augmented compared to control (about 50 %, *P* < 0.05) in presence of VitD alone or in samples pre-treated with VitD. These data demonstrate that VitD is able to explain its effects through VDR receptor signaling through genomic action.

### p53, pan-Ras, Ki67, and c-Myc Analysis in FSEC

**Fig. 4:**
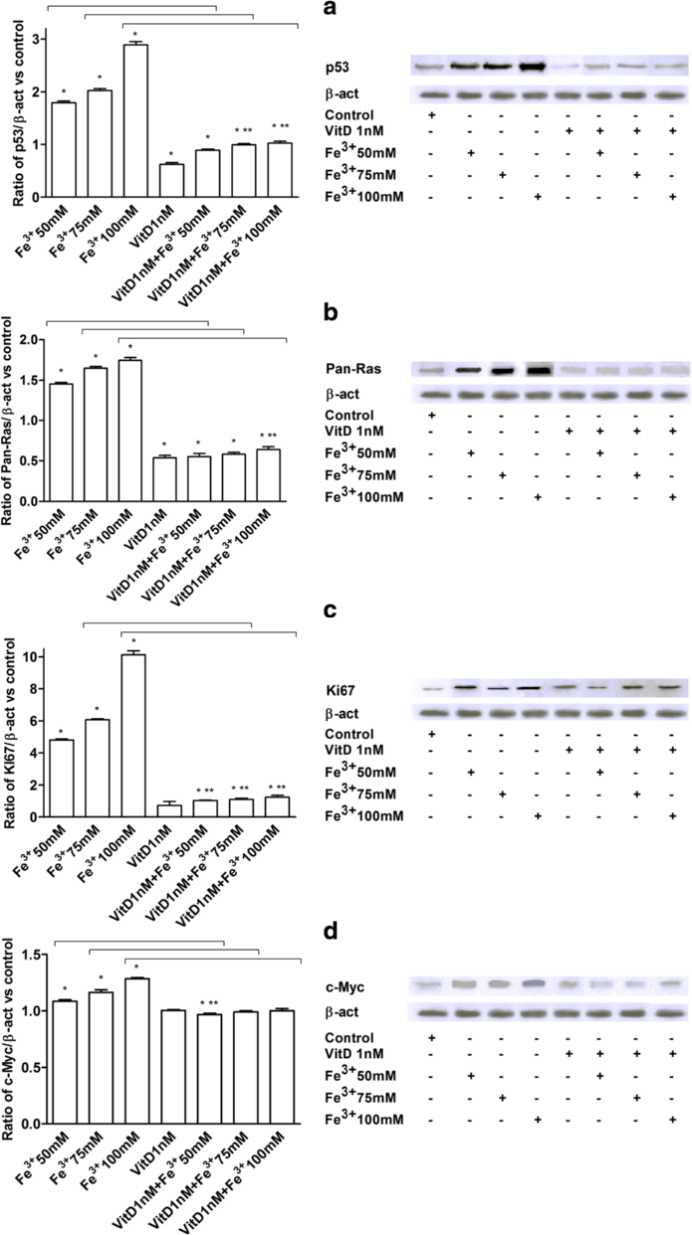
Western Blot and densitometric analysis of p53 (**a**), Pan-Ras (**b**), Ki67 (**c**) and c-Myc (**d**). Protein extracts were analyzed by immunoblotting with specific antibodies against the indicated proteins. Data are expressed as means of 5 technical replicates performed on FSEC. * *P* < 0.05 *vs* control; ** *P* < 0.05 *vs* VitD; arrows indicate *P* < 0.05 between Fe^3+^ alone and VitD + Fe^3+^

**Fig. 5:**
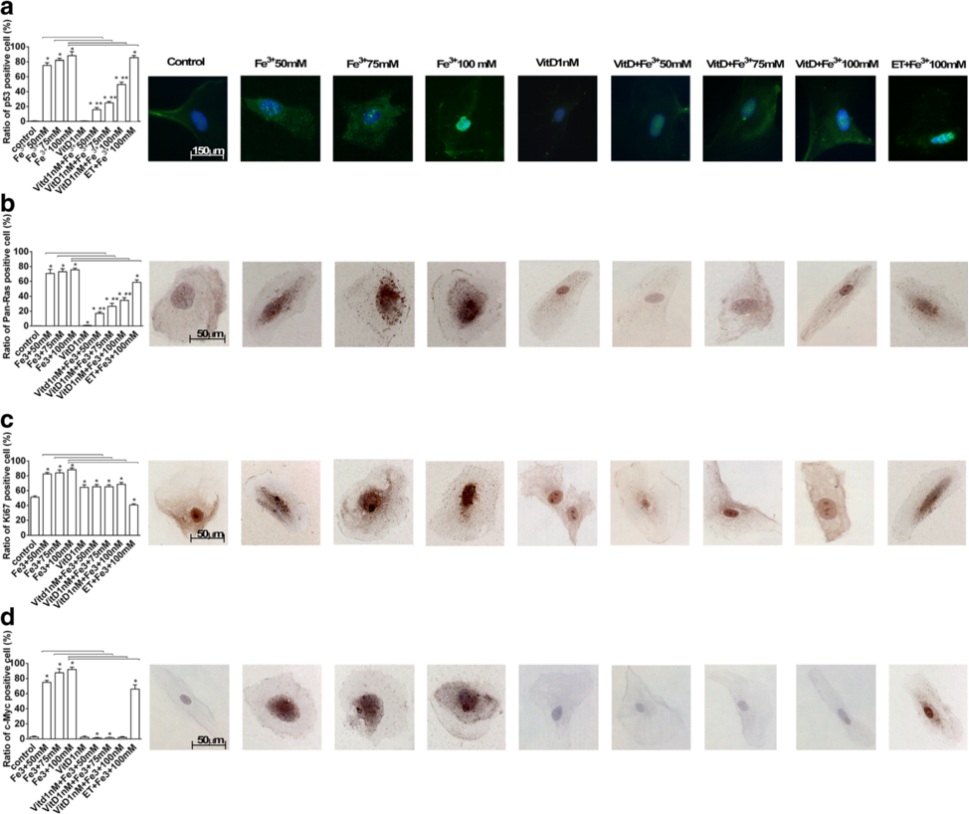
p53 (**a**), Pan-Ras (**b**), Ki67 (**c**) and c-Myc (**d**) analysis by immunofluorescence (panel A) or immunocytochemistry (panel B-D). On the left the score of positive cells, counted in 12 different areas, and on the right the representative pictures, obtained through microscopy at original magnification of X40, are reported. The ratio is reported as mean (SD) (%) of positive cells of 5 technical replicates. The scale reported in the first column on the right is to be considered valid for the same antibody. * *P* < 0.05 *vs* control; ** *P* < 0.05 *vs* VitD; arrows indicate *P* < 0.05 between Fe^3+^ alone and VitD + Fe^3+^

p53, pan-Ras, Ki67, and c-Myc expressions were investigated in FSEC treated with Fe^3+^ alone and after pre-treatment with VitD by Western blotting (Fig. 4) and immunofluorescence or immunocytochemistry (Fig. 5). The effects of VitD alone on these pathways were also tested in the same protocols (Figs. 4 and 5). In presence of Fe^3+^ p53, pan-Ras, Ki67, and c-Myc activations were observed in a dose-dependent manner, and the maximum effects were observed with Fe^3+^ 100 mM after 6 days of treatment (2.88 ± 1.06; 1.74 ± 1.03; 10.13 ± 1.23;1.28 ± 1.01 ratio of activation, respectively) compared to control values by Western blotting (Fig. 4). Similar data were also observed in immunofluorescence (p53) and immunocytochemistry experiments (pan-Ras, Ki67, c-Myc) performed in FSECs (Fig. 5). The pre-treatment with VitD for 6 days was able to counteract p53, pan-Ras, Ki67, and c-Myc activations (Figs. 4 and 5): these effects were clearer in samples treated with Fe^3+^ 100 mM (64 %; 42 %; 87 %; 22 % of reduction, respectively by Western Blot analysis). In addition the effects of VitD alone were also tested and didn’t reveal a significant activation of tumoral markers (*P* < 0.05) compared to control values.

## Discussion

It has been demonstrated that the site of origin of most high grade serous ovarian cancers is the fallopian tube secretory epithelial cells [33]. Recent studies hypothesized the role of retrograde menstruation-derived Fe^3+^ in increasing risk of ovarian cancer [25], due to a severe oxidative injury induced by iron [26, 34, 35]. Fimbria in the pouch of Douglas is exposed to generated from hemolysis of erythrocytes by pelvic macrophage during retrograde menstruation, a common physiologic event in all menstruating women [25]. In addition, Seidman [35] showed the presence of mucosal iron in fallopian tubes in advanced-grade pelvic serous carcinoma [36]. In a previous work, Lattuada et al. [26] demonstrates in FSEC the involvement of Fe^3+^ in carcinogenic changes using Fe^3+^ at highly doses [22, 30]. This study demonstrates for the first time that VitD plays an important role in preventing Fe^3+^-dependent oxidative stress in cultured fimbrial cells. Experiments were performed using 1 nM VitD. Experiments using Fe^3+^ have been preceded by a dose-response study showing that the best effect on cell viability was obtained with 1 nM VitD and the optimum range is between 0.1 and 10 nM. This is the concentration range considered physiologically and clinically attainable in humans [29]. The great decrease observed in cell viability after 100 nM VitD administration depends primarily on saturation of the intracellular pathways controlling viability. This effect is still higher than the control. The beneficial effect of VitD consists in a significant decrease of oxidative state showed by a significant decrease in ROS production in culture supernatants. However the ability of VitD to block oxidative injury has been tested only when this substance has been administered before Fe^3+^.

These findings support previous studies in which the relationship between low VitD serum concentration and overall survival rate of patients with ovarian cancer has been demonstrated [10]. Moreover, the important role of severe VitD deficiency in more aggressive course of ovarian cancer has been described [6, 35–37]. The effects of VitD have been observed in FSEC with a high grade of culture purity (PAX-8 positivity) along with the activation of VDR. For this reason mechanisms underlying protective effects of VitD may be hypothesized to have genomic origin.

p53, pan-Ras, Ki67 and c-Myc has been analyzed in FSEC treated with VitD alone or before Fe^3+^, to clarify the protective mechanism activated by VitD. A novel finding is represented by the demonstration that pre-treatment with VitD is able to significantly counteract tumoral biomarkers activation, suggesting the inhibition of epithelial cell transformation. Thus our findings indicate that VitD prevented the activation of p53, pan-Ras, Ki67, and c-Myc. On the contrary, Fe^3+^ alone was able to mimic in FSEC, through these tumoral biomarkers, the carcinogenic changes typical of serous ovarian cancer.

As concerns Fe^3+^ concentration adopted in this study, it must be considered that cellular iron homeostasis is regulated by cytosolic regulatory proteins that bound structural elements (iron-response elements) present in the messenger RNA of some major proteins such as transferrin receptor and ferritin. For this reason it is difficult to quantify the plasmatic concentration of free Fe^3+^. However, in this study, it has been chosen to use a Fe^3+^ concentration similar to that found in endometriotic cysts [30].

The importance of these data is remarkable since the Fe^3+^ concentration used in these experiments may induce carcinogenic changes as reported by many studies [26, 38, 39].

## Conclusions

The results described herein highlight that VitD exerts protective effects against Fe^3+^-related oxidative stress in cultured FSEC. The discussed results could be relevant in the light of the use of serum VitD levels assessment to promote VitD supplementation or to adjust therapeutic strategies in ovarian cancer patients.
